# Factors affecting the use of herbal products in patients with Irritable Bowel Syndrome and their results: case–control study

**DOI:** 10.1186/s12876-022-02125-z

**Published:** 2022-02-04

**Authors:** Emin Gemcioglu, Nuray Yılmaz Cakmak, Salih Baser, Servet Kocaoz, Osman Ersoy

**Affiliations:** 1 Department of Internal Medicine, Ankara City Hospital, Ankara, 06100 Turkey; 2 Department of Internal Medicine, Yıldırım Beyazıt University School of Medicine, Ankara, Turkey; 3 Department of General Surgery, Ankara City Hospital, Ankara, Turkey; 4 Department of Gastroenterology, Yıldırım Beyazıt University School of Medicine, Ankara, Turkey

**Keywords:** Irritable bowel syndrome, Herbal therapy, Audio-visual media

## Abstract

**Background:**

Irritable bowel syndrome (IBS) is a functional bowel disease that is characterized by abdominal pain, discomfort, and changes in the frequency and form of stool without any organic pathology. In this study, the factors that affect the herbal treatment choices of IBS patients and their results were investigated.

**Methods:**

Included in the study were 248 IBS patients who were over the age of 18. A questionnaire that comprised 25 questions was applied to the participants. Survey questions were asked to the participants regarding their age, place of birth, gender, educational status, demographic details, social standing, socioeconomic status and job, place of residence, and marital status. In addition, The participants were asked about which IBS symptoms they had, from whom they had received the recommendation for use of herbal products, whether the media had an effect on their selection of herbal products, and whether they had benefited from herbal products.

**Results:**

It was observed that 41.1% of the patients with IBS who participated in the study used herbal medicine, 9.8% of whom used them regularly. It was found that the IBS patients participating in the study made their decision to use herbal products mostly based on the recommendations that they were given by acquaintances (57%) and the media (34%). When the patients were evaluated according to their gender, IBS was found to be more common in unemployed women who had a low level of education, while it was more common in working men (*p* = 0.015, *P* < 0.001, respectively). The IBS patients who were single used more herbal products that those who were married (*P* = 0.036). While the use of herbal herbs and oils was predominant in patients whose recommendation content comprised the media/internet and acquaintances, the herbal treatment content recommended by healthcare professionals consisted of traditional treatments and mixtures (*P* = 0.012). It was determined that a higher percentage of those who used herbal treatments lived in city centers when compared to those who did not (*P* < 0.001). In addition, it was determined that patients with constipation used herbal products more than those without (*P* < 0.001). Among the IBS patients, those who had diarrhea and those who were receiving medical treatment preferred to use significantly less herbal products (*P* = 0.007 and *P* = 0.041, respectively). It was found that the patients who visited the Gastroenterology Outpatient Clinic mostly used herbal therapy, while those who visited a family doctor used herbal therapy the least (*P* = 0.029 and *P* < 0.001, respectively).

**Conclusion:**

The IBS patients revealed whose recommendations they followed when purchasing herbal products, which of the products they preferred, and how useful/beneficial they felt that these products were. In this regard, the addition of training curricula related to herbal treatment for professional healthcare workers will further raise awareness on this topic.

## Introduction

Irritable bowel syndrome (IBS) is a functional bowel disease that is characterized by abdominal pain, discomfort, and changes in the frequency and form of stool without any organic pathology. IBS is the most common gastrointestinal system disease in patients who have visited internal and surgical outpatient clinics [1, 2]. Its prevalence is increasing among individuals between the ages of 30 and 50. The disease is twice as common in women than in men worldwide, while this rate is 3 times more in Turkey [3, 4]. High socioeconomic status and modern living conditions have been identified as risks for IBS development [5]. The chronic nature of IBS and the lack of an effective treatment cause a loss of workforce and worsening of the quality of life of patients [6]. IBS physiopathology includes genetic factors, intestinal microbiota, impaired immune system regulation, changes in intestinal permeability, and bowel-brain interaction [7]. Rome IV criteria are used in the diagnosis of IBS [8]. The main goal of treatment is to relieve symptoms. Patients frequently use alternative and complementary treatments due to the chronic nature of the symptoms, inadequate treatment response, and medication side effects in IBS [9]. Complementary and alternative treatments for IBS include hypnosis, acupuncture, cognitive behavioral therapy, yoga, and herbal medicine. Herbal medicine has a therapeutic effect as well as side effects in IBS [9, 10]. Herbal products are usually used uncontrolled and without consulting a doctor or determining the correct dosage. A previous study found that the source of information of patients taking herbal therapy comprised other patients who had the same complaints, the media, the internet, their friends and relatives, spice merchants, and herbalists [11]. In this study, the factors that affect the herbal treatment choices of IBS patients and their results were investigated.

## Materials and methods

In this study, patients who applied to the Internal Medicine and Gastroenterology Outpatient Clinic of a tertiary referral hospital, between January and April 2018, were evaluated. Among these patients, those whose clinical situations were in line with the ROME IV criteria were included in the study [8]. Those with malignancy and inflammatory bowel disease, who were pregnant, or had any alarm symptoms (recent onset and rapid exacerbation of complaints, onset of symptoms after the age of 50, weight loss, fever, anemia, bloody defecation, symptoms awakening from sleep at night, family history of gastrointestinal cancer, antibiotics use, and travel history to countries with risk of infection) were excluded from the study. A questionnaire was applied to the patients who were accepted for participation in the study (Appendix).

Before the questionnaire was administered, the patients were informed about the aim of the study, verbal consent was obtained, and it was stated that they could withdraw from the study at any stage. The questionnaire included questions about the sociodemographic characteristics of the participants, IBS, and herbal therapy.

### Statistical analysis

Statistical analysis of the data was performed using IBM SPSS Statistics 25.0 (IBM Corp., Armonk, NY, USA). The compliance of continuous numerical variables to normal distribution was tested using the Kolmogorov–Smirnov test. Among the descriptive statistics, the continuous numerical variables were presented as the mean ± standard deviation or median [minimum (min) – maximum (max)], while the categorical variables were presented as the frequency and percentage. While the difference between the groups in terms of the mean was evaluated using the student t test, the difference in terms of the median was evaluated using the Mann–Whitney U test. The categorical variables were analyzed using the Pearson chi square test. Unless otherwise stated, *P* < 0.05 was considered as statistically significant.

## Results

The number of patients included in the study was 248, and of these, 139 were women (56%) and 109 (44%) were men. The mean age of the patients was 43.6 ± 13.5 years; the mean age of the women was 44.5 ± 13.0 years, while the mean age of the men was 42.4 ± 14 years. Only 41.1% of the patients were receiving herbal treatment, 9.8% of whom were using it regularly. Of the IBS patients, 46% were primary and secondary school graduates, 23.4% were high school graduates, and 20.2% were undergraduates. It was found that IBS was significantly more common in women who had low education than the men with the same educational background (*P* = 0.015). Considering their occupations, IBS was found to be significantly more common in women who did not work than in men who did (*P* < 0.001). Considering their marital status, 74.6% of the patients were married and 25.4% were single. It was found that the single patients used significantly more herbal products for IBS (*P* = 0.036). The patients were mostly residents of district centers (46%). The sociodemographic data of the patients are given in Table 1. The most common complaints among the patients on admission were abdominal distension (79%), constipation (70.6%), and abdominal pain (66.9%) (Table 2). The mean duration of time that the patients had typical IBS symptoms was 5 months (min: 3, max: 43). The number of outpatient clinic visits and the imaging characteristics are given in Table 3. While 87.1% of the patients did not use the medication that was given to them regularly, 1.6% used it regularly, and 11.3% did not receive any medical treatment (Table 4). Of the patients, 102 were receiving herbal therapy (41.1%), while 146 (58.9%) were not. Those who received herbal therapy mostly preferred a single herb (12.5%) or daily traditional solutions (12.5%), such as mint-lemon and linden. Apart from these, they used vegetable oils (10.1%) and herb mixtures from herbalists (5%), respectively. The patients did not have any detailed information about the ingredients of the mixtures that they bought from most of the spice merchants. In addition, traditional treatments generally vary depending on the society but are known by everyone, such as drinking mint and lemon tea for stomach pain and nausea, using fennel tea for abdominal pain, and using probiotic yogurt that contains olive oil and flaxseed for constipation. Other herbal herbs, concoctions (such as tea mixed with mint, fennel, ginger, chamomile, and cinnamon), and oils are herbal products that are usually prepared by the spice merchants. The information and suggestions that the patients received for the use of herbal therapy were mostly from their neighbors, relatives, and friends (57%). Other sources included the media/internet (34%) and health professionals (9%). While the use of herbal herbs and oils was predominant in patients whose content was the media/internet and acquaintances, the herbal treatment content recommended by healthcare professionals consisted of traditional treatments and mixtures (*P* = 0.012). While 23.5% of the patients found the herbal treatment to be ineffective, 22.5% stated that they found it to be weakly effective, and 54% found it to be effective (moderate and very effective). Moreover, 1 patient stated that s/he experienced life-threatening side effects. While 9.8% of the patients used herbal therapy regularly, 47.06% did not (Table 5). There was no significant relationship between the professions of the patients and the herbal therapy that they used (*P* = 0.774). There was no significant difference in terms of female and male gender in terms of herbal treatment usage (*P* = 0.573). It was found that those who received herbal therapy mostly lived in city centers (*P* < 0.001). Those suffering from diarrhea among the IBS patients preferred to use significantly fewer herbal products (*P* = 0.007). Patients who received medical treatment used herbal therapy less than the others (*P* = 0.041). It was found that the patients who visited the Gastroenterology Outpatient Clinic mostly used herbal therapy, while the patients who visited a family doctor used herbal therapy the least (*P* = 0.029, *P* < 0.001, respectively) (Table 6). There was no significant relationship between the types of herbal therapies used by the patients and the side effects of these treatments (*P* = 0.392) (Table 7). Patients with constipation preferred to use herbal therapy more than the patients without constipation (*P* < 0.001) (Table 8).

**Table 1:** Sociodemographic data of the patients

Sociodemographic characteristics	Frequency (n = 248)	Percentage (%)
Gender		
Women	139	56
Men	109	44
Educational status		
Illiterate	13	5.2
Literate	13	5.2
Primary and secondary school graduate	114	46
High-school graduate	58	23.4
Undergraduate	50	20.2
Profession		
Housewife/retired	121	48.8
White-collar	41	16.5
Blue-collar	71	28.6
Student	15	6
Marital status		
Married	185	74.6
Single	63	25.4

**Table 2:** Complaints of the patients at the time of admission

Complaint	Frequency (n = 248)	Percentage (%)
Abdominal pain	166	66.9
Abdominal distention	196	79
Intestinal gas	163	65.7
Nausea	112	45.2
Vomiting	38	15.3
Diarrhea	73	29.4
Constipation	175	70.6
Constipation + diarrhea	58	23.4

**Table 3:** Symptom duration, outpatient clinic admission, and imaging characteristics of the patients receiving herbal therapy

Variable	Median (min–max)
Symptoms duration	5 (3–43) months
Number of visits to a family doctor	4 (0–12)
Number of visits to a gastroenterology outpatient clinic	1 (0–4)
Number of colonoscopies	1 (0–3)
Number of imagings (ultrasound, CT and MRI)	2 (1–3)

**Table 4:** Medical treatment and features related to the herbal therapy use of the patients

	Frequency (n = 248)	Percentage (%)
How often patients receive medical treatment		
Regularly	4	1.6
Irregularly	216	87.1
Does not receive any	28	11.3
Herbal use		
Using	102	41.1
Not using	146	58.9

**Table 5:** Characteristics of herbal treatment

	Frequency (n = 102)	Percentage (%)
Content		
Herbal herbs	31	30.4
Oils	25	24.5
Mixtures	15	14.7
Traditional solution	31	30.4
Recommendation source for herbal use		
Media/internet	35	34
Neighbors/relatives/friend	58	57
Health professionals	9	9
Herbal effect		
No effect	24	23.5
Weak	23	22.5
Medium	26	25.5
Good	22	21.6
Very good	7	6.9
Herbal side effect		
No effect	89	87.2
Weak	8	7.8
Medium	2	2
Strong	2	2
Life-threatening	1	1
Frequency of herbal use		
Regular	10	9.8
Often	44	43.14
Occasionally/rarely	48	47.06

**Table 6:** Comparison of the patients receiving herbal therapy and those not receiving herbal therapy in terms of symptom duration, outpatient clinic admission and imaging

Variable	Using herbal therapy (n = 102) Median (min–max)	Not using herbal therapy (n = 146) Median (min–max)	*P* value
Symptom duration	5 (3–43)	5 (3–43)	0.881
Number of visits to a family doctor	3 (0–12)	4 (0–10)	< 0.001
Number of visits to a gastroenterology outpatient clinic	1 (0–4)	0 (0–2)	0.029
Number of colonoscopies	1 (0–3)	1 (0–3)	0.811
Number of imagings	2 (1–7)	2 (1–5)	0.159

**Table 7:** Comparison of the types of herbal therapy and their side effects

Herbal content	No/weak herbal effect n, (%)	Has an herbal effect n, (%)	Total n, (%)	*P* value
Herbal herbs	16, (51.6)	15, (48.4)	31, (100)	0.392
Oils	14, (56)	11, (44)	25, (100)	
Mixtures	6, (40)	9, (60)	15, (100)	
Traditional solution	11, (35.5)	20, (64.5)	31, (100)

**Table 8:** Comparison of the patients receiving herbal therapy and those not receiving herbal therapy in terms of their characteristics

Variable	Using herbal therapy n, (%)	Not using herbal therapy n, (%)	Total n, (%)	*P* value
Single	33, (52.4)	30, (47.6)	63, (100)	0.036
Married	69, (37.3)	116, (62.7)	185, (100)	
City center	64, (62.1)	39, (37.9)	103, (100)	< 0.001
District	28, (24.6)	86, (75.4)	114, (100)	
Town/village	10, (32.3)	21, (67.7)	31, (100)	
Women	55, (39.6)	84, (60.4)	139, (100)	0.573
Men	47, (43.1)	62, (56.9)	109, (100)	
Employed	44, (39.3)	68, (60.7)	112, (100)	0.592
Unemployed	58, (42.6)	78, (57.4)	136, (100)	
Receiving medical treatment	96, (43.6)	124, (56.4)	220, (100)	0.041
Not receiving medical treatment	6, (21.4)	22, (78.6)	28, (100)	
Has constipation	86, (49.1)	89, (50.9)	175, (100)	< 0.001
Does not have constipation	16, (21.9)	57, (78.1)	73, (100)	

## Discussion

IBS is a chronic functional disorder of the gastrointestinal tract that is characterized by abdominal pain, abdominal distension, diarrhea, and episodes of constipation, and it has a high prevalence and diverse etiology worldwide [9, 12]. The current approach in the treatment of this syndrome is to reduce the symptoms rather than eliminate the underlying causes of the disease. Although there are various pharmacological treatment options, due to the limited effectiveness, high cost, and side effects of these treatments, the use of herbal therapy has become more common worldwide. In the current study, patients who received no medical treatment, lived in city centers, and had a relatively low socioeconomic and cultural levels tended to receive herbal therapy. This study was not a community-based study, and the selection of those who wanted to participate in the survey from the patients who had applied to the outpatient clinics was considered as the reason why the gender difference was not evident in both the frequency of IBS and the use of herbal.

The Chinese herbal products that have been used in East Asia for thousands of years are examples of this treatment [13]. Acupuncture, which is a type of traditional medicine that has been mentioned frequently in the media, can also be used with herbal therapies, and in a study by Yan et al., the administration of herbal therapy together with acupuncture was found to be safe and effective [14]. As it is around the world, some of the patients herein who participated in the study tended to use herbs and traditional methods. Although the herbal therapies used were partially effective with a single use, it has been reported that the combinations created from these products can also be used, and this may be more beneficial [9]. It is also believed that many factors, such as the regular use of these products and the regular maintenance of medical treatment by the patient, affect the benefits obtained from herbal products. It was believed herein that regular treatment and follow-up can have a positive effect on IBS and herbal treatment, as in every disease. These products, which are also popular with IBS patients, have gained vast attention in the written, visual, and verbal media. Although most of the participants in the current study obtained their herbal product recommendations from their neighbors, relatives, and friends, the influence of the media in the selection of herbal products was also quite high. The patients obtained advisory information for these herbal therapies from the media rather than from health professionals. The effect/side effect profile of herbal therapies is not clear, as different herbal therapies may show different effects on patients, and these unpredictable effects have a wide spectrum, from acute renal failure to toxic hepatitis. It is obvious that the information given about these herbal products covered in the media may not be objective, non-commercial, or completely correct [15]. Unfortunately, because of commercial concerns or hearsay information about these products, for which safety and efficacy criteria are not clear, patients may be misled, and these products may cause various side effects or less beneficial effects than expected. A study by Munyaradzi et al. evaluated the effects of advertisements for herbal therapies on African societies and found that the headlines used in media-based advertisements and interesting statements about the product, even if not scientific information, affected the decisions of the patients [16]. It is thought that the herbal products used by IBS patients, which may be beneficial/harmful or have no effect in various situations, may deviate from their purpose due to the biased/unbiased promotion of the media and bilateral relations in daily life. The fact that the majority of patients who participated in the current study did not receive herbal therapy may be a result of these concerns.

Madisch A et al. reported in their study that 54% of primary care physicians were satisfied with the success of spasmolytic treatment, and 75% were satisfied with phyto-therapeutic agents and probiotics [17].

## Conclusion

For IBS patients to use herbal products correctly and appropriately, they should be provided unbiased information about these herbal products. Relatives, neighbors, etc. In this regard, the addition of training curricula related to herbal treatment for professional healthcare workers will further raise awareness and it will increase the conscious use of herbal medicine in the treatment of IBS.

## Data Availability

The datasets used and/or analyzed during the current study are available from the corresponding author on reasonable request.

## Appendix

The questionnaire about evaluatıon of knowledge and attıtude of persons wıth gastroenterologıcal complaınts:

## Declarations

### Helsinki declaration

The authors declared that this study is in accordance with the Helsinki Declaration.
